# Cardiac Fibroblasts and the Extracellular Matrix in Regenerative and Nonregenerative Hearts

**DOI:** 10.3390/jcdd6030029

**Published:** 2019-08-20

**Authors:** Luis Hortells, Anne Katrine Z. Johansen, Katherine E. Yutzey

**Affiliations:** The Heart Institute, Cincinnati Children’s Medical Center, Cincinnati, OH 45229, USA

**Keywords:** cardiac fibroblast, extracellular matrix, cardiac regeneration

## Abstract

During the postnatal period in mammals, the heart undergoes significant remodeling and cardiac cells progressively lose their embryonic characteristics. At the same time, notable changes in the extracellular matrix (ECM) composition occur with a reduction in the components considered facilitators of cellular proliferation, including fibronectin and periostin, and an increase in collagen fiber organization. Not much is known about the postnatal cardiac fibroblast which is responsible for producing the majority of the ECM, but during the days after birth, mammalian hearts can regenerate after injury with only a transient scar formation. This phenomenon has also been described in adult urodeles and teleosts, but relatively little is known about their cardiac fibroblasts or ECM composition. Here, we review the pre-existing knowledge about cardiac fibroblasts and the ECM during the postnatal period in mammals as well as in regenerative environments.

## 1. Introduction

A regeneration continuum exists from invertebrates to vertebrates—planarians can regrow all tissues, amphibians and fish can regenerate limbs and hearts, while adult mammals are capable of wound healing, but in general do not regenerate body parts [1]. In the adult mammalian heart, a cardiac injury results in the formation of a permanent fibrotic scar in the absence of regeneration. In contrast, young mammals (fetal or neonatal), urodeles, and some teleosts are capable of cardiac regeneration, with minimal scar formation [2]. In the past two decades, cardiac regeneration research has been focused on cardiomyocytes—which actively de-differentiate and proliferate in regenerating species [3] but not in adult mammals [4]. Substantially less research effort has been devoted to the cardiac fibroblast, which is essential in the wound healing response and generation of the extracellular matrix (ECM). In the absence of regeneration, a permanent fibrotic scar is formed, which has deleterious effects on normal cardiac function. Subsequent progressive cardiac fibrosis is a clinical manifestation of hypertrophic cardiomyopathy, dilated cardiomyopathy, ischemic heart disease, and heart failure, correlating with disease severity and a poor prognosis [5,6,7]. Cardiac fibroblasts and the ECM are also critical regulators of cardiac organogenesis [8,9], and thus their interactions with cardiomyocytes, immune cells, and endothelial cells may contribute to the ability to undergo adequate regeneration. Here, we review and focus on what is known about the cardiac fibroblast and the ECM in regenerating and non-regenerating species to highlight components that may facilitate cardiac regeneration.

## 2. Cardiac Fibroblasts and the Extracellular Matrix

During cardiac organogenesis in vertebrates, cardiac fibroblasts arise from mesothelial pro-epicardial cells that form at the venous pole and migrate over the looped heart tube [10]. A subset of these pro-epicardial derived cells undergo epithelial–mesenchymal transition and migrate into the myocardial wall to give rise to coronary smooth muscle cells and cardiac fibroblasts [11,12,13,14,15]. This process is driven by, in part, transforming growth factor-β (TGF-β), bone morphogenic protein (BMP), Wingless-related integration site (Wnt), and retinoic acid signaling [13,16] and has been extensively reviewed elsewhere [17,18]. The expression of the basic helix–loop–helix (bHLH) transcription factor 21 (Tcf21) and the receptor tyrosine kinase platelet derived growth factor receptor alpha (PDGFR-α) are necessary for the formation of cardiac fibroblasts from pro-epicardial cells by epithelial–mesenchymal transition [9,17]. In the adult mouse heart, cardiac fibroblasts constitute more than 20% of the non-cardiomyocyte fraction [19] and exist mostly as single cells within the interstitial space outside the basement membrane [20].

Under homeostatic conditions, cardiac fibroblasts are considered ‘quiescent’ cells with a low proliferation and ECM turnover [21]. However, in response to certain growth factors, hypoxia and/or tissue injury, cardiac fibroblasts rapidly transition to an activated cell type that synthesizes abundant ECM proteins, mediating wound contracture and cell-communication both within the tissue and remotely. Recent studies have shown that resident cardiac fibroblasts, as opposed to bone marrow-derived cells, are the principal source of activated fibroblasts and myofibroblasts in mammals and zebrafish in response to injury [22,23].

In general, three basic phases of wound healing have been described: inflammation, proliferation, and remodeling [24]. In the heart, the apoptosis of cardiomyocytes, triggered by stress, hypoxia or nutrient deprivation, stimulates the innate immune system and the influx of inflammatory mediators. Neutrophils, monocytes, and/or tissue resident macrophages [25] infiltrate and eliminate dead cells, while releasing cytokines, that in part mediate the proliferation of cardiac fibroblasts and promote ECM synthesis [26]. In the proliferative phase, fibroblasts synthesize abundant quantities of ECM and restructure the collagen fibers. In the final remodeling phase, the scar tissue evolves structurally, both in its composition and tensile strength. In the absence of a suitable replacement of scar tissue with functional cardiomyocytes, as occurs in regenerating species such as urodeles and teleosts, the normal organ architecture is lost indefinitely, and cardiac contractility is significantly compromised.

Fibroblasts are the major cell type contributing to the synthesis of the ECM, which surrounds the cardiomyocytes and acts as a scaffold, maintaining the myocardial tissue architecture. In addition to the mechanical and structural support that the ECM provides, it also participates in cell signaling through growth factor interactions and integrins, modulating biological processes such as proliferation, apoptosis, migration, and differentiation [27,28] and thus has critical functions during cardiac embryogenesis and injury healing [29,30,31,32].

Constituents of the cardiac ECM include major structural components, such as fibrillar collagens (Col), fibronectin (FN), proteoglycans, and glycosaminoglycans. Additionally, there are also non-structural matricellular proteins such as periostin (Postn), which participate in critical signal transduction pathways [32,33]. During ECM remodeling, matrix metalloproteinases (MMPs) degrade the ECM, which can be inhibited by tissue inhibitors of metalloproteinases (TIMPs) [34,35]. Recent studies have shown that modulation of the ECM can affect cardiomyocyte proliferation and/or migration. While it is evident that cardiac regenerative potential is related to the ability of cardiomyocytes to de-differentiate and proliferate, it is becoming increasing clear that scarring and fibrosis impede regeneration (discussed further below). To gain further insight into the potential fine-tuned regulation of cardiac regeneration by the cardiac fibroblast and ECM, we will review what is known in regenerating (urodeles, teleosts, and mammalian embryos and neonates) and nonregenerating species (adult mammals).

## 3. Contributions of Cardiac Fibroblasts and the ECM to Cardiac Regeneration in Urodeles

Different members of the amphibian family of the urodeles, such as the axolotl (*Ambystoma mexicanum*) and the newt (*Notophthalmus viridescens*), have a remarkable capacity for regeneration, including the heart [36,37,38,39,40,41]. The newt and axolotl heart consist of two atria and one ventricle. The ventricular myocardium of the axolotl or the newt heart can be rapidly regenerated back to normal even after amputation of 30–50% [36,39,40]. In an uninjured adult newt heart, cardiomyocytes are not proliferative; however, in response to injury, they de-differentiate and undergo mitosis to restore lost tissue [42]. During the first week after injury, a transient fibrin/collagen clot deposition occurs to protect the heart from cardiac rupture, which is degraded by MMPs as cardiomyocytes proliferate and replenish the lost tissue [43,44,45]. Depending on the extent of the injury, the heart will be regenerated with no evidence of scarring within 60–90 days [43].

Several studies have examined the gene regulation governing cardiomyocyte proliferation in amphibians [46], however less research has been conducted on the relative contributions of cardiac fibroblasts and the ECM. In a microarray analysis of ventricular samples from newts at 3-, 7- and 14-days post-injury, MMPs, as well as the ECM genes, *FN* and *Tenascin-C* (*TNC*) were initially found to be the most highly upregulated genes [44]. However, this study analyzed whole ventricular tissue as opposed to cardiac fibroblasts, thus obscuring direct contributions of fibroblasts. Cryoinjury of the axolotl ventricle results in a robust induction of α-smooth muscle actin expression, a myofibroblast marker associated with enhanced collagen synthesis and fibrosis [47]. Here it was shown that that the immune response is essential in the process of wound healing and that the axolotl heart regenerates via a transient collagenous network [47]. Depletion of macrophages induced cardiac fibroblast activation resulting in a modified ECM that inhibited cardiac regeneration [47]. The total number of fibroblasts was however not affected, as determined indirectly by unchanged vimentin (a fibroblast marker) expression. Together, these studies provide evidence that cardiac fibroblasts play a role in the regeneration of the urodele heart, however a detailed examination is currently lacking.

Relatively little is known about the cardiac ECM in the adult urodele homeostatic heart. The expression of Col3 [45] and the absence of TNC have been described [44]. A more comprehensive analysis of the cardiac ECM in the regenerating newt heart has however revealed the importance of the ECM components TNC, hyaluronic acid (HA), and FN in mediating regeneration [44]. By 3 days post-injury, TNC is upregulated around the injured area, followed by epicardial expression at 7 days and around the fibrous clot at 14 days post-injury. HA is expressed around the clot and the epicardium 7 days after injury. The expression of HA and TNC is maintained up to day 35 after injury, and later becomes restricted to the regenerative areas. FN is moderately expressed at the beginning of the healing process, followed by upregulation at 21 days post-injury, lasting up to day 49 post-injury.

To directly assess the involvement of the spatiotemporally expressed ECM proteins on cardiomyocyte regeneration, primary newt cardiomyocytes were plated on culture dishes coated with TNC, HA or FN, and only TNC was found to substantially increase cardiomyocyte proliferation [44]. In a cryoinjury model of infarction in the axolotl heart, depletion of macrophages reduced the expression of FN and TNC while increasing the expression of collagen I [47]. As a result, there was excessive collagen deposition and fibrosis, despite no observed differences in cardiomyocyte proliferation. These data confirm that there is interplay among fibroblasts, the ECM, cardiomyocytes, and macrophages in mediating the regenerative response. In salamanders, activation of TNC expression, accumulation of FN and HA to induce cell de-differentiation [48,49,50,51], and induction of MMPs in lysing the provisional scar [52], have also been implicated in limb regeneration. These data together suggest that similar processes in the limb and the heart related to ECM composition and cellular regulation facilitate regeneration in multiple contexts.

## 4. Contributions of Cardiac Fibroblasts and the ECM to Cardiac Regeneration in Teleosts

The adult teleost heart consists of one atrium and one ventricle. In a similar fashion to the urodeles, some teleost fishes can regenerate different parts of their body, including the heart. The zebrafish (*Danio rerio*) has been used for a long time to study the mechanisms of both cardiac development and regeneration [53]. In contrast to adult mammalian hearts, some teleosts, such as the zebrafish, are able to regenerate their hearts with minimal scar formation [54]. Surgical removal of ~20% of the adult zebrafish heart initially results in blood clot formation, which promotes the deposition of collagen and fibrins, generating a provisional scar. After 9 days, new cardiomyocytes generated from proliferation of existing cardiomyocytes migrate into and around the clot, and 30 days after amputation, virtually complete regeneration of the heart occurs with little evidence of scar formation [54,55].

In the zebrafish, pre-existing and, to a lesser extent, newly-derived endocardial fibroblasts contribute to collagen synthesis after injury [56]. Ablation of *col1a1*-expressing cells in the injured zebrafish heart did not affect regeneration at 35 days post injury, although a significant reduction in the number of proliferating cardiomyocytes was observed [56]. This suggests that both fibroblasts and the ECM are required for cardiomyocyte proliferation and subsequent regeneration in the zebrafish. Lineage tracing studies in the zebrafish demonstrate that Tcf21-expressing cells give rise to the epicardial cells and cardiac fibroblasts [57]. Although these cells are found within the trabecular myocardium, they are located in close proximity to the epicardium [56,57,58] and are not dispersed throughout the myocardial wall or in the same quantities as what is reported in the mouse heart [9]. Sequencing analysis of these cardiac fibroblasts shows that they express the signature mammalian cardiac fibroblast genes *Tcf21*, *Col1a1a*, *Col1a2*, *Col5a1*, *Dcn*, *Postn*, *Sox9a,* and *Thbs1b* [56]. Single-cell sequencing analysis of a small population (31 single cells) of cardiac fibroblasts reveals cellular heterogeneity amongst these cells [58]. In response to ventricular cryoinjury, Postn-positive activated fibroblasts [23] emerge and surround the injured area, rapidly proliferating at 7 and 14 days post-injury, which decreases significantly after 21 days [56]. As is observed in the mouse [23], Postn-positive cells are derived from Tcf21-expressing cardiac fibroblasts [56]. Sequencing analysis of the Postn-positive cells showed increased expression of secreted proteins (~14%), as well as the ECM proteins Postn, FN, thrombospondins, and collagens [56], similar to gene expression profiles of mammalian cardiac fibroblasts post injury [59]. Many of the signaling genes identified in this study were members of the Wnt family, which are critical for heart development, regeneration, and fibrosis [60]. In addition, several matrix metalloproteases (MMPs) increased in expression at 7 days post injury, which could be involved in remodeling and degrading the ECM [56]. As cardiomyocytes proliferate and regenerate the injured tissue, activated cardiac fibroblasts (Postn-positive) cells revert back to a quiescent cell type with a similar expression profile to uninjured cells. However, these cells uniquely upregulate the expression of several collagens [56], suggesting that, although they may resemble their original identity, the injury has modified their expression profile.

However, not all teleosts are able to regenerate. *Astyanax mexicanus*, is a single fish species that exists in two forms—the surface and the cave fish. Interestingly, during evolution the cave form lost the ability to regenerate the heart after injury [61]. In both surface and cave fish subspecies, apical resection resulted in the formation of a collagen-rich scar. In the surface fish, this was replaced by newly regenerated muscle within ~2 months, whereas, in the cave fish, a permanent fibrotic scar was observed. Unexpectedly, cardiomyocyte proliferation after injury was similar between the two subspecies. However, proliferation of non-myocyte cells was significantly increased in the cave fish. RNA-sequencing analysis identified upregulation of immune and fibroblast response genes in the cave fish compared to the surface fish. *Caveolin* and *lrrc10* were upregulated in the surface fish and *caveolin1* has previously been found to be essential for zebrafish heart regeneration [58]. The Medaka fish is another example of a teleost that does not regenerate following cardiac apical resection [62]. In this model, there was also no increase in cardiomyocyte proliferation detected but there was extensive scar formation after injury.

Further analysis of cardiac fibroblasts, cellular crosstalk mechanisms, and the ECM in these models is needed to better define their functions in regeneration. However, while cardiomyocyte de-differentiation and proliferation are critical for cardiac regeneration, fibroblast activation, proliferation, and re-differentiation back towards a quiescent cell type, also appear to be integral to the regenerative process.

From studies of cardiac development, we know that in the zebrafish embryo, cardiomyocyte progenitors migrate along a basal membrane that contains FN, laminin, and sulfated proteoglycans [63,64,65,66]. FN is initially deposited in the lateral plate mesoderm and later in the midline between the endoderm and the endocardial cell layers. Loss of FN or the proteoglycan syndecan-2 results in heart malformations due to abnormal migration of myocardial progenitors. This suggests that these ECM proteins could have an important role in cardiomyocyte migration after injury and hence in cardiac regeneration capacity as has been shown in mouse models [67]. On the other hand, loss of different laminins (1, β1 or γ1) individually did not disrupt heart tube formation. Recently, Garcia-Puig and colleagues performed proteomic analysis of decellularized adult zebrafish hearts both in homeostasis and after injury [68]. Their data show that the most abundant ECM proteins in the sham operated zebrafish heart are FN 1b, collagens (6, 1, 4, and 5) and fibrinogens (fga, fgg, fgb) [68].

One of the more abundant ECM proteins present after different types of cardiac injury in the zebrafish is FN [69]. Even without directly affecting cardiomyocyte proliferation, deficiency of this protein impairs cardiac regeneration [69]. The regenerative capacity of the zebrafish heart has been shown to be dependent on TGF-β signaling, whereby inhibition of this pathway reduced total fibroblasts and subsequent collagen deposition [70]. In a proteomic analysis of the zebrafish cardiac ECM 7 days after injury [68], the most differentially expressed proteins were fibrinogen, FN1, and Postn. At 60 days after injury, samples of the ECM were obtained from regenerated areas and control animals, and expressional analysis confirmed resolution of the provisional scar.

Taken together, the results from studies of urodeles and teleosts support the importance of FN in the cardiac regeneration process. The role of FN in regeneration is still not clear. However, based on the available data in cardiac development, it would be interesting to evaluate the influence of FN on cardiomyocyte migration to the injured area. On the other hand, TGF-β seems to have a basic role in regeneration, probably activating fibroblasts to generate the provisional scar, but in a highly regulated fashion, because excessive activation of this pathway has been shown to promote fibrosis instead of regeneration, as has been shown in salamanders [47]. The necessity of specific activation pathways and ECM remodeling by the cardiac fibroblasts agrees with the generation of a temporary scar and its resolution in regenerative species versus formation of a permanent scar in non-regenerative systems.

## 5. Contributions of Cardiac Fibroblasts and the ECM to Cardiac Regeneration in Mammals

The mammalian heart consists of two atria and two ventricles. The advent of modern genetic tools to identify and trace fibroblasts has dramatically accelerated the understanding of cardiac fibroblasts and fibrosis in mice. Cardiac fibroblasts constitute less than 20% of the total cells in the mouse heart [19] and are interspersed uniformly throughout the myocardial wall [71]. During development, single cell sequencing analysis has identified fibroblast-like cells at embryonic day (E) 11.5 in the right ventricle and at E14.5 throughout the heart [72]. The relative numbers of fibroblast-like cells remain stable at E18.5, postnatal (P) day 0 and P3. In a different study, the quantity of cardiac fibroblasts was significantly increased between P1 and P15, with further increases in the adult heart [73]. In a recent analysis, ~10% of cardiac fibroblasts in the left ventricle were proliferating at P2, which increased to ~20% at P4 and P7 [21]. The rate of proliferating fibroblasts was dramatically reduced at P7, similar to the time when cardiomyocytes stop proliferating [21].

The neonatal mouse heart at P1 is able to regenerate after apical resection [4] or myocardial infarction [74], but this potential is lost by P7. The regeneration process is similar to that observed in the zebrafish—robust inflammation with extensive fibrosis, which is rapidly replenished with healthy myocardium [4,74]. As is observed in the zebrafish, the newly formed cardiomyocytes are derived from pre-existing cardiomyocytes [4]. In larger mammals, neonatal pigs are also able to regenerate after a myocardial infarction, but the capacity to regenerate is lost within the first 2–3 days after birth [75,76]. Indeed, significant scar formation was observed in pigs with a myocardial infarction at 3 days old, compared with minimal scar formation observed in pigs operated at 2 days old [75]. RNA-sequencing analysis of the hearts at 1 and 7 days post myocardial infarction showed little evidence of a fibrotic signature in the young pigs (P2) that are able to regenerate [75]. However, an in-depth analysis of the cardiac fibroblasts in these samples is lacking. In humans, a single case report presented a newborn child who had a severe myocardial infarction due to a coronary occlusion [77]. Remarkably, within weeks after thrombolytic therapy, there was recovery of normal cardiac function, and echocardiograms at 1 year of age were indistinguishable from healthy hearts. While this case study demonstrates that early cardiac regeneration may be conserved across mammals, no data are available on cardiac fibroblasts and ECM in human infant hearts after injury. Studies in neonatal mice have demonstrated that the ability to regenerate is dependent on cardiomyocyte proliferation. For example, inhibition of cardiomyocyte proliferation using cardiomyocyte-specific microRNA (miR)-195 (a member of the miR-15 family) overexpressing mice resulted in extensive cardiac fibrosis with no regeneration after myocardial infarction at P1 [74]. These data suggest that proliferation of pre-existing cardiomyocytes is necessary for regeneration. However, it does not exclude the possibility that the fibroblasts and the ECM could modulate cardiomyocyte proliferation.

In early gestation in mammals (including sheep, rats, mice, pigs, monkeys, and humans), the skin heals without scarring, a concept known as scarless fetal healing [78]. In examining the differences between fetal scarless healing and adult wound repair, fetal wounds were found to have less inflammation, increased hyaluronic acid related to cell movement, altered fibrillar collagen ratios, and lower expression of TGF-β1 and β2 [78]. Cardiac fibroblasts synthesize and deposit ECM proteins, and thus differences in fetal versus adult fibroblasts may underlie the regenerative potential within a given tissue. Furthermore, fibroblasts secrete cytokines that can enhance the regenerative potential, by, for example, promoting cardiomyocyte proliferation and angiogenesis [79].

In a phenotypic and transcriptomic analysis of human cardiac fibroblasts, fetal fibroblasts were smaller and had an increased cell turnover compared with adult fibroblasts [80]. Gene ontology analysis revealed enrichment in ephrin-receptor, notch, IL-8 signaling, axonal guidance signaling, hepatic fibrosis, cardiovascular system development, and organismal and embryonic development genes in fetal cardiac fibroblasts. In contrast, adult cardiac fibroblast genes were enriched in gene ontology terms including IL-6 signaling, hepatic fibrosis, immune cell trafficking, tissue development, and organismal development. In adult human cardiac fibroblasts, fibroblast growth factor 7, integrin subunit alpha 8, crystallin alpha B, elastin, and osteoprotegerin were highly expressed. In fetal human cardiac fibroblasts, interleukin-1B, Postn, heparin-binding epidermal growth factor-like, and vascular cell adhesion protein were highly expressed [80]. In a comparison of embryonic and adult mouse cardiac fibroblasts, higher expression of *FN*, *TNC*, collagen genes, and *Postn* were found in embryonic samples [81]. This expression profile was shown to be associated with enhanced cardiomyocyte proliferation providing evidence that both the fibroblasts and the ECM can regulate cardiac regeneration [81].

Postn is a secreted matricellular protein involved in wound healing, which is abundantly expressed in response to injury in activated fibroblasts [82]. Lineage tracing of Postn-expressing cells marks all myofibroblasts (α-smooth muscle actin expressing cells) and almost all these cells are derived from resident cardiac fibroblasts marked by Tcf21 [23]. Gene expression profiling of these Postn-expressing cells identified *Col1a1*, *Col1a2*, *Adam12*, *Ctgf, TNC*, *Wisp1*, *Elastin,* and *Fibrillin* as highly expressed genes, amongst others [23]. In contrast to zebrafish, where ablation of cardiac fibroblasts results in less cardiac regeneration [56], in adult mice, ablation of activated fibroblasts (Postn+) after the period of acute injury response reduces fibrosis and improves cardiac function [83]. More cardiomyocytes were observed within the scar area [83], suggesting that the altered ECM may be more amenable to regeneration, although this was not assessed. Alternatively, the Postn+ cardiac fibroblasts may provide signals to the cardiomyocytes that contribute to loss of regenerative ability, but this has not yet been demonstrated. In contrast, ablation of Postn-expressing cells during or immediately after myocardial infarction resulted in greater lethality due to ventricular wall rupture and demonstrates that these cells are necessary in forming the initial protective scar [23]. The finding that the ablation of Postn+ cells after the initial scar formation is protective may be attributed to the lack of matrifibrocytes derived from the Postn+ cells, which would contribute to mature scar formation in adults [59]. Matrifibrocytes have a unique gene signature resembling bone, connective tissue, and cartilage, including expression of *Comp*, *Chad*, and *Cilp2* [59].

Molecular regulatory mechanisms of cardiac fibrosis and control of fibroblast numbers in mice has also been reported. For example, Wnt/β-catenin signaling, which is critical during cardiac development and is increased in diseased hearts [84], was found to be necessary for pathologic fibroblast activation and proliferation in response to transverse aortic constriction (TAC) [60] and cardiac ischemia [85]. The loss of Wnt/β-catenin signaling in resident and activated fibroblasts in the heart reduced interstitial fibrosis and collagen expression without affecting the number of fibroblasts or activated fibroblasts [60]. Moreover, in a model of pathological hypertrophy, ablation of Wnt/β-catenin signaling in cardiac fibroblasts also reduced cardiomyocyte hypertrophy and improved cardiac function [60]. TGF-β, which is essential for the formation of cardiac fibroblasts, is also critical in the transition of a fibroblast towards a myofibroblast [86]. Furthermore, the development of fibrosis in hypertrophic cardiomyopathy is dependent on profibrotic TGF-β ligand expression in cardiomyocytes and signaling in cardiac fibroblasts [87,88]. Specifically, genetic deletion of TGF-β receptors 1 and 2 or Smad2/3 in cardiac fibroblasts reduced fibrosis and cardiac hypertrophy in response to TAC [87,88]. Mechanistically, TGF-β receptor-depleted fibroblasts failed to express the myofibroblast marker, α-smooth muscle actin within their stress fibers, and fibroblast proliferation and expansion was significantly impaired [88]. In addition, ablation of Hsp47, a stress-induced collagen chaperone that is necessary for collagen synthesis and secretion, in activated cardiac fibroblasts results in ineffective scar formation and cardiac rupture after myocardial infarction by reducing the expression of fibrillar collagens and fibroblast proliferation [89]. Furthermore, upon chronic pressure overload stimulation, cardiac fibroblast-specific Hsp47 knockout mice fail to mount a fibrotic response and cardiomyocyte hypertrophy is significantly reduced [89]

Taken together, these studies show that cardiac fibroblasts are necessary for adequate scar formation and that the subsequent ECM profile influences cardiomyocyte proliferation and hypertrophy. However, as cardiomyocyte proliferation is inherently low in adult hearts [90], there is limited scar resolution and fibroblasts transition towards a matrifibrocyte, which results in mature scar formation [59] reducing cardiac compliance. The ablation of matrifibrocytes may promote a ‘softer’ scar tissue that is amenable to cardiomyocyte expansion, that over time will allow further regeneration.

The mammalian cardiac ECM is composed of different members of the families of proteoglycans, collagens, elastin, fibrillin, TNC, FN, and laminins [91]. During development, several of these proteins have been described to be essential for a normal cardiac formation. The independent genetic ablation of hyaluronan synthase [92], perlecan [93], versican [94], Col1 [95], Col4 [96], Col5 [97], and FN [98] showed embryonic lethality with abnormal cardiac embryogenesis. In addition, ablation of aggrecan [99], hapln1 [100], glypican-3 [101], Col2 [102], fibulin1 [103], and fibrillin1 [104] has been shown to be lethal neo/perinatally. Postn ablation has showed moderate neonatal lethality, mostly related to cardiac valve malformations [105].

Little is known about the healthy postnatal ECM in mammalian hearts. One study showed the differences among fetal, postnatal, and adult cardiac ECM in rats using mass spectrometry after decellularization [106], finding a 6.5-fold reduction of FN (which is considered necessary in developmental pre-collagenous ECM). FN, heparin-binding epidermal growth factor-like growth factor, and fibrillar collagens have also been shown to have paracrine effects inducing mouse cardiomyocyte proliferation in vitro through B1-integrin [81]. Certain ECM proteins that have been associated with increased cardiomyocyte proliferation in lower vertebrates, such as TNC in the newt, seem to have the opposite effect in adult mammalian hearts after injury, increasing fibrosis [107]. A study using TNC-KO mice reported delayed myofibroblast and macrophage recruitment necessary in the healing response, suggesting different roles in the cardiac fibrotic process [108]. Similarly, FN has been implicated in embryonic cardiomyocyte migration in lower mammals and neonates and its absence impairs regeneration even in the presence of proliferative cardiomyocytes [47]. On the other hand, specific ablation of FN in cardiac fibroblasts is partially protective against fibrosis in adult mice with cardiac injury [109], probably through impeding fibroblast migration and excessive ECM deposition.

The previously mentioned study on postnatal development [106] also showed a 4-fold increase in the content of Col1 in the fetal compared to the adult cardiac ECM. Interestingly, Col3 and Col5 were not detected in fetal or postnatal hearts, while they were detected in the adult heart ECM. Postn and fibrillin-2, on the other hand, were almost undetectable in the adult hearts but were detected in the total ECM in the fetal hearts. Postn is reactivated after injury in the adult mouse and is required for fibroblast activation and scarring in adult mammals [23]. However, the role for Postn in the postnatal heart is unclear. Studies have shown that exogenous delivery of Postn [23] after myocardial infarction in mice can induce myocardial proliferation and promote cardiac repair [110], but at the same time, other studies fail to see changes in cardiomyocyte number or cell cycling activity with Postn ablation or overexpression [111]

Other components of the postnatal cardiac ECM have also been implicated in the regulation of cardiomyocyte proliferation and induction of cell cycle arrest soon after birth. In mice, the neonatal (P1) ECM can promote increased cardiomyocyte proliferation in vitro compared to P7 ECM [112]. In the same study MMPs, in particular the gelatinase MMP9, inhibited the proliferation induced by the ECM extracted from P1 mice. In addition, one of the targets of MMP9, agrin, was more highly expressed at P1, compared with P7, and injection of agrin promoted cardiac regeneration in adult mice post-myocardial infarction [112]. The authors suggest that this effect can happen through a mechanism involving the disassembly of the dystrophin–glycoprotein complex, together with hippo and extracellular regulated kinase-mediated signaling, in cardiomyocytes.

From a biomechanical standpoint, there is increasing evidence that ECM stiffness may be a major factor in cardiomyocyte cell cycle arrest [113]. In a transcriptomic analysis comparing P1 and P2 mouse hearts, major differences were found in cytoskeletal and ECM genes. To further confirm the influence of the ECM on the regeneration capacity, an in vivo loss of ECM stiffness experiment showed that cardiac regenerative capacity is increased with a less stiff ECM in P2 hearts [114]. Others have shown the influence of the rigidity of the cellular substrate on cardiomyocyte differentiation, cytokinesis, and karyokinesis. Yahlom-Ronen et al. reported that in more rigid microenvironments, proliferative cardiomyocytes tend to generate binucleated cardiomyocytes in vivo and in vitro. In contrast, in less rigid environments, proliferating cardiomyocytes undergo cytokinesis, generating two mononucleated cells [113]. These studies demonstrate that, in addition to possible paracrine effects, acquiring a stiffer ECM structure through increasing the collagen content and its organization likely contributes to cardiomyocyte cell cycle arrest.

**Figure 1:**
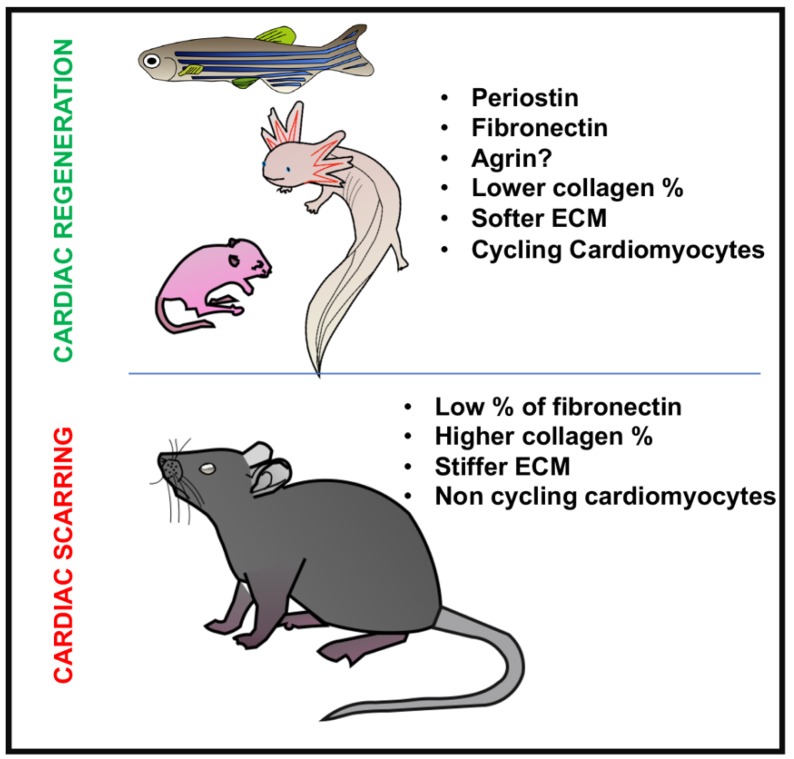
Diagram showing the main differences in ECM and cardiac fibroblasts between regenerative (zebrafish, axolotl, neonatal mice) and non-regenerative animals (adult mice).

Taken together, these studies show that composition of the ECM, consisting of less collagen, with more FN and Postn, facilitates heart regeneration (Table 1). Also, it seems that in mammalian hearts, once the adult ECM is established and the cardiomyocytes have exited the cell cycle, the upregulation of “repairing” ECM constituents, such as FN or TNC, is not enough by itself to induce proliferation, and as a result, a collagenous stiff scar is formed. Only the administration of agrin has recently been shown to be capable to induce cardiomyocyte dedifferentiation and cell cycle re-entry, but how this works is still to be clarified. Finally, all these models provide evidence for specific ECM characteristics that facilitate or enhance cardiac regeneration in teleosts, urodeles, and neonatal rodents (Figure 1). Additional studies are needed to determine if manipulation of the ECM can promote cardiac regeneration in larger mammals including pigs or humans after birth. Alternatively, direct reprogramming of cardiac fibroblasts has also been proposed as a mechanism to enhance cardiac regeneration/repair and could be used therapeutically in combination with manipulation of the cellular microenvironment [115]. Recent reports show neonatal cardiac regeneration up to postnatal day 3 in pigs, but ECM components, cellular contributions, and crosstalk mechanisms that underlie loss of regenerative capacity are not well-understood. Thus, further investigation is needed in order to completely define the role and potential therapeutic applications of cardiac fibroblasts and ECM in animal models similar to humans.

## 6. Conclusions and Future Directions

The regeneration process in the hearts of such different animals as amphibians, fish, and neonatal mammals seems in essence, similar. Even the first steps of the healing process in adult mammals mirror those in the regenerative species. With this in mind, the reasons why some animals can resolve the scar initially formed to maintain heart function after injury, while others maintain the scar compromising long-term heart function, are still not fully understood. In this review we have compared the ECM dynamics and the fibroblast activity in cardiac development, homeostasis, and after injury. It has been shown that even in the presence of proliferative cardiomyocytes, proper ECM formation by the cardiac fibroblasts is required for regeneration [69]. This confirms that the regeneration process is more complex than just induction of cardiomyocyte proliferation. Rather, it is a perfect symphony of different factors that begins with the adequate crosstalk between macrophages and cardiac fibroblasts that induce deposition of a pro-regenerative ECM and leads to the migration of proliferative cardiomyocytes to repopulate the affected area. One of the main obstacles to cardiac regeneration in adult mammals is the complexity and stiffness of the mature cardiac ECM which is linked to cardiomyocyte hypertrophy and cell cycle arrest [114]. Mature cardiomyocytes are highly specialized cells that are refractory to dedifferentiation and proliferation within the context of the highly structured contractile myocardium. Thus, adult cardiac injury in mammals is characterized by formation of a permanent scar. There is increasing evidence that targeted inactivation of cardiac fibroblasts or ECM maturation could be useful to improve the outcome after a myocardial infarction. This way, a more moderate deposition of ECM by the cardiac fibroblasts could be cardioprotective, and at the same time, reduce the scar tissue area, improving myocardial function. In the same direction, increased cardiomyocyte proliferation and, in consequence, less cardiomyopathy, could be induced in the presence of a more primordial ECM profile as is present in the neonatal heart. In fact, it has been shown that cardiac regeneration in the zebrafish requires the formation of a provisional clot/scar by activated fibroblasts that will become progressively less active as the new cardiomyocytes populate the injured area [56].

Recently, important advances in understanding postnatal cardiac fibroblasts and the ECM that they generate have been made, but there are still important gaps in our knowledge of the architects of myocardial maturation and the structure itself. We consider that understanding the characteristics and behavior of cardiac fibroblasts and their crosstalk with cardiomyocytes and other cells through the ECM and other secreted proteins, from birth to postnatal day 7, is imperative to generate new therapeutic approaches like heart patches [124]. Finally, we should not forget that these data proceed mainly from small animal models, but if we really want to move forward in the treatment of human heart fibrosis, we need to understand the postnatal period of heart development and regeneration in larger mammals such as the pig and of course, in our own species.

## Figures and Tables

**Table 1:** Extracellular matrix (ECM) proteins in regenerative and nonregenerative hearts.

	Urodeles	Teleost	Postnatal Mammals	Adult Mammals
**Collagen 1**	Constituent of the permanent scar formed after macrophage ablation [47].	Increased expression in cardiac fibroblasts after injury [56].	Less basal content than in adult hearts [106].	More expression than in the postnatal heart [106]. Constituent of the scar after injury [116].
**Collagen 3**	Present [45].		Not detected [106].	Present [106].
**Collagen 5**		Increased RNA expression after injury [68].	Not detected [106].	Present [106].
**Collagen 7**		Present after resolution of transient [68].		
**Collagen 8**		Expressed by fibroblasts once the transient scar is resolved [56].		Present. Induces fibrosis and myofibroblast activation in pressure overload models [117].
**Collagen 10**		Expressed by fibroblasts once the transient scar is resolved [56].		
**Collagen11A1** **Collagen11A2**		Expressed by activated fibroblasts after injury [56]. Expressed by fibroblasts once the transient scar is resolved [56].		
**Collagen 12**		Expressed by activated fibroblasts after injury [118].		
**Collagen 13**		Expressed by fibroblasts once the transient scar is resolved [56].		
**Fibronectin**	Overexpressed 21 days after injury (before cardiomyocyte migration) [44].	Expressed in basal conditions [68]. Highly upregulated after injury, prior to cardiomyocyte migration [69]. Ablation induces scar formation and blocks regeneration [69].	Expressed in basal conditions [106].	Reduced expression in basal conditions [106]. Specific ablation in fibroblasts reduces fibrosis [109].
**Tenascin C**	No basal expression but highly expressed 3 days after injury [44]. Reduced expression in scar after macrophage ablation [45].			Not expressed in normal conditions. Induces fibrosis after injury promoting myofibroblast migration [107].
**Periostin**		Expressed in cardiac fibroblasts after injury [56].	Expressed in basal conditions [106,119].	Not expressed in basal conditions [106]. Required for cardiac fibroblast activation and scar formation after injury [23,120,121].
**Hyaluronic acid**	Highly expressed 3 days after injury [44].		Expressed during embryogenesis [122].	Expressed in basal conditions [122]. Actively formed after injury [123].
**Agrin**			Expressed in basal conditions [112].	Not expressed in basal conditions [112].
**Fibrillin-2**			Expressed in basal conditions [106].	Not expressed in basal conditions [106].

## Abbreviations

ECM	Extracellular matrix
TGF-β	Transforming growth factor-β
BMP	Bone morphogenic protein
Wnt	Wingless-related integration site
b-HLH	Basic helix-loop-helix
Tcf21	Transcription factor 21
PDGFR-α	Platelet-derived growth factor receptor alpha
FN	Fibronectin
Postn	Periostin
MMPs	Matrix metalloproteinases
HA	Hyaluronic acid
TNC	Tenascin-C
Fg	Fibrinogen
Col	Collagen
